# Diagnostic Accuracy Studies in Radiology: How to Recognize and Address Potential Sources of Bias

**DOI:** 10.1155/2021/5801662

**Published:** 2021-09-06

**Authors:** Athanasios Pavlou, Robert M. Kurtz, Jae W. Song

**Affiliations:** ^1^St. Vincent's Medical Center, Bridgeport, CT, USA; ^2^Frank H. Netter MD School of Medicine, North Haven, CT, USA; ^3^Hospital of the University of Pennsylvania, Philadelphia, PA, USA

## Abstract

Accuracy is an important parameter of a diagnostic test. Studies that attempt to determine a test's accuracy can suffer from various forms of bias. As radiology is a diagnostic specialty, many radiologists may design a diagnostic accuracy study or review one to understand how it may apply to their practice. Radiologists also frequently serve as consultants to other physicians regarding the selection of the most appropriate diagnostic exams. In these roles, understanding how to critically appraise the literature is important for all radiologists. The purpose of this review is to provide a framework for evaluating potential sources of study design biases that are found in diagnostic accuracy studies and to explain their impact on sensitivity and specificity estimates. To help the reader understand these biases, we also present examples from the radiology literature.

## 1. Introduction

The accuracy of a diagnostic test refers to how well a test can correctly identify a specific disease. Therefore, it is a crucial parameter to consider when making a decision to perform that test in a clinical setting. Inaccurate diagnostic tests can lead to over- or undertreatment, inflated healthcare costs, and potentially patient harm [1]. Diagnostic accuracy studies attempt to evaluate a test's performance by comparing it to a gold standard. These studies can suffer from biases (e.g., spectrum bias and verification bias) that are different from those affecting studies designed to test the efficacy of therapeutic interventions. Awareness of these biases and how they can impact diagnostic accuracy measures is important. Several studies have quantitatively shown that specific biases can lead to an overestimation or underestimation of accuracy measures [2, 3]. Given that diagnostic accuracy studies help experts and policymakers to create guidelines and establish standard-of-care measures [4], it is imperative that readers be aware of these biases and how they can be addressed.

As practitioners of a diagnostic specialty, it is important for radiologists to understand how to appraise diagnostic accuracy studies. Radiologists are frequently consulted by other physicians on which imaging test to order for specific indications and serve to educate and inform others about current standards of care for the diagnostic work-up of many patients. In the era of evidence-based medicine, radiologists are encouraged to keep up with the literature as well as know how to appraise the quality of a diagnostic accuracy study. Moreover, it is equally important to know how applicable the results of a particular diagnostic accuracy study are to the radiologist's own clinical practice [5].

Guidelines and checklists often serve as useful tools to help one be comprehensive and achieve consistency. As such, the Cochrane Collaboration and Agency for Healthcare Research and Quality has recommended the use of checklists such as the Quality Assessment of Diagnostic Accuracy Studies 2 (QUADAS-2) tool [6]. This tool helps to assess the risk of bias in diagnostic studies and is organized into 4 key domains. These domains include evaluating aspects of study design related to (1) patient selection, (2) the index test, (3) reference standard, and the (4) flow and timing of subjects in a study [7]. Within each domain are specific types of study design biases that should be considered.

In this paper, we use the QUADAS-2 framework to review the study design biases within each domain (see Table 1). We will also present examples from the radiology literature.

## 2. Basic Concepts

**Figure 1:**
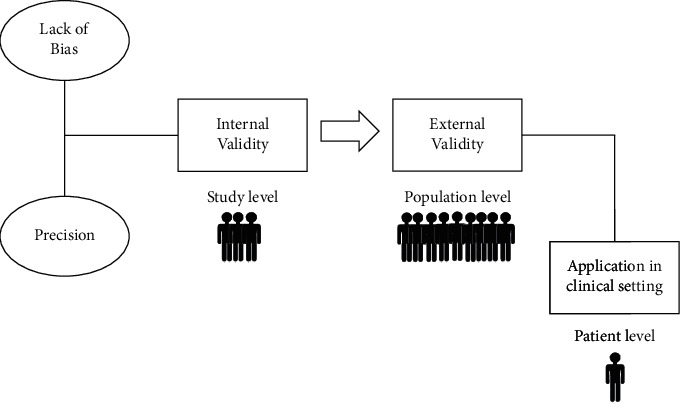
Internal and external validity. Precision and lack of bias dictate the internal validity of the study. External validity refers to the process of applying the study results from the study level to the population level. Radiologists can use these results in their own clinical practice for management of individual patients.

The framework for developing a research question in evidence-based medicine follows the PICO model. In diagnostic accuracy studies, PICO stands for P (population), I (index test), C (comparator or reference standard), and O (outcomes). A diagnostic accuracy study compares the index test (the test under investigation) with an established reference test on a specific population and provides outcomes for comparison [8]. The degree to which the outcomes of the study represent true findings among similar individuals outside the study is determined by the validity. There are two main types of validity: internal and external (see Figure 1) [9].

### 2.1. Internal Validity

**Figure 2:**
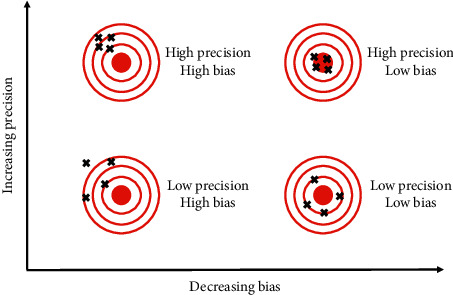
Precision and bias. Increasing precision reduces the random error and decreasing bias is equivalent to decreasing systematic error. The higher the precision and the lower the bias, the higher the internal validity of the study. Adapted from ELife, 7, e35718, Brandmaier, A. M. et al., Assessing reliability in neuroimaging research through intraclass effect decomposition (ICED) (2018) (modified) [10].

The extent to which the observed results are not due to methodological errors is defined as internal validity. The internal validity of a study can be threatened by bias and imprecision (see Figure 2). Bias is considered to be any systematic deviation of an estimate from the true value. If a diagnostic accuracy study suffers from bias, its sensitivity and/or specificity will be consistently under- or overestimated compared to the true value. This means that the error introduced by bias will not balance out upon repetition. Imprecision is the random error that occurs with multiple estimates of a parameter and refers to how far these estimates are from each other, not how far they are from the true value. Because of the random deviation of the estimates towards opposite directions, repetition will eventually balance out this error [11].

### 2.2. External Validity

External validity examines whether the findings of a study can be generalized to the population level. If the study's sample is representative of the target population, the results of the study can be generalized to the population from which the sample was drawn and even beyond that to other similar populations. This is especially important as it determines whether the results of the study can be applied in daily clinical practice [12].

Applicability is also an important consideration when evaluating diagnostic accuray studies. Careful evaluation of the PIC (Population-Index-Reference) parameters of a study will help determine the extent of applicability of a study to a reader's clinical practice. The patient demographics, selection and use of the index test, and test interpretation should be compared between the study and the reader's practice. To allow for this comparison, it is vital that diagnostic accuracy studies report their methods with completeness and transparency, preferably using standardized checklists such as the Standards for the Reporting of Diagnostic Accuracy studies (STARD) [13].

Using the PIC framework will help the reader assess the external validity as well as gain insight into the applicability of the study. For assessment of internal validity, critically appraising the study design using a four-domain framework is suggested [11]. We now review specific sources of study design biases using the QUADAS-2 framework.

## 3. Domain 1: Patient Selection

The goal of sampling is to ensure that the sample group is representative of the population of interest. The results of the study are contingent on the studied sample. Thus, sampling methods are a critical part of a study design. Participants should ideally be recruited from a population in a process that ensures no over- or underrepresentation of certain subpopulations [14].

### 3.1. Sampling Definition and Methods

Sampling is the process of selecting a group of study subjects from the target population. There are two main categories of sampling methods: probability and nonprobability sampling.

In probability sampling methods, all eligible subjects in the target population have equal chances to be selected (e.g., random sampling). The challenge with this type of sampling method is that it requires the presence of a comprehensive list or registry of all eligible patients in the target population, from which the subjects are randomly chosen using, for instance, a random number generator [14]. As such registries are rarely available in practice, clinical studies more frequently use nonprobability sampling [15].

In nonprobability sampling methods, the sample is selected in a process that does not guarantee equal chances to be selected for each eligible subject in the target population. An example of nonprobability sampling is convenience sampling, where patients are selected only based on accessibility and availability. The selection process for convenience sampling can lead to over- or underrepresentation of certain population attributes and therefore decreases the generalizability of the study results (sampling bias). A special form of convenience sampling, commonly used in clinical research, is consecutive sampling. In this sampling method, for a specified period of time, every subject who meets the predefined inclusion and exclusion criteria is recruited for the study. This sampling method prevents the researchers from “picking and choosing” subjects [15]. Analysis of 31 published meta-analyses showed that nonconsecutive sampling tended to overestimate the diagnostic accuracy of the test by 50% compared to consecutive sampling in diagnostic accuracy studies [16].

The effect of consecutive over nonconsecutive sampling can be seen in a study evaluating deep venous thrombosis (DVT) of the lower extremities. Kline et al. recruited subjects using a consecutive method to compare the diagnostic accuracy of emergency clinician-performed compression ultrasonography for DVT of the lower extremities against whole-leg venous ultrasonography and reported a sensitivity of 70% and specificity of 89% [17]. By contrast, other studies on the same topic reported almost perfect diagnostic accuracy (sensitivity: 100% and specificity: 91.8–100%) using a nonconsecutive sample. These higher accuracy measures could be due to excluding complex cases, excluding patients who may be difficult to perform ultrasound, or excluding ambiguous results [18, 19].

### 3.2. Spectrum Bias

Spectrum bias is commonly used to describe the variation in test performance across patient subgroups. Studies that utilize a limited portion of the patient spectrum can be affected by this type of bias. For example, a study that includes only high-risk patients may provide different diagnostic accuracy estimates compared to a study that includes only low-risk patients, as the test performance varies in different populations [20, 21].

An obvious source of spectrum bias is a patient selection method that leads to a sample that is not representative of the target population. Local referral practices can also remove cases from the initial distribution, narrow the spectrum of patients, and lead to bias [11]. Understanding spectrum bias is important as it can prohibit the generalization of the results from the studied sample to a wider population, especially when studying heterogeneous populations. It has been suggested that “spectrum effect” is a more appropriate term, as the estimate from a narrow spectrum of patients is valid for this specific subgroup [21].

An example of how diagnostic accuracy measurements can be influenced by the patient spectrum is seen in a meta-analysis that studied the accuracy of magnetic resonance imaging (MRI) to detect silicone breast implant rupture. The authors found that the diagnostic accuracy of MRI in studies that included patients with symptoms of implant rupture was 14 times higher compared to studies that included only asymptomatic patients and two times higher compared to studies that used both symptomatic and asymptomatic patients (screening sample) [2].

### 3.3. Case-Control and Cross-Sectional Study Design

**Figure 3:**
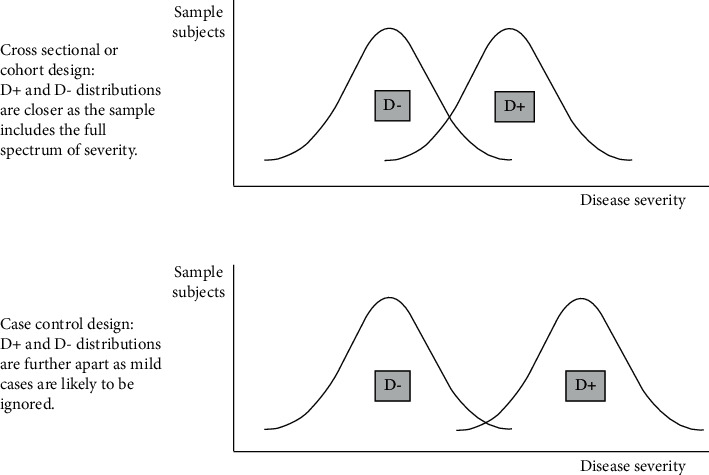
Cross-sectional study design minimizes spectrum bias as cases and controls are not sampled separately from the target population. D refers to disease status with D+ meaning disease is present and D-patients meaning disease is absent.

In diagnostic accuracy studies, based on the way subjects are recruited, the study design is usually a case-control, cross-sectional, or cohort study design. In case-control designs, patients are sampled separately from controls, which introduces spectrum bias. This is because patients tend to be “the sickest of the sick,” which leads to sensitivity overestimation, and controls tend to be the “healthiest of the healthy,” which leads to specificity overestimation (see Figure 3). In cross-sectional and cohort designs, patients and controls are sampled together from a population based on the presence of a characteristic regardless of the presence of disease [3, 22]. In a study by Lijmer et al., which reviewed 184 diagnostic accuracy studies for design-related bias, case-control designs tended to overestimate the diagnostic performance of the test by threefold compared to studies with cohort design [3].

An area in radiology where the difference between case-control and cohort has been studied is Artificial Intelligence (AI). As noted by Park [23], utilizing a case-control design for the clinical validation of AI algorithms forces a binary distinction of outcomes that does not accurately represent real-world situations, where disease-simulating conditions and comorbidities may be present. As a result, the diagnostic performance of an AI algorithm may be inflated, and consequently, the generalization of study results to real-world practice may be problematic. Nevertheless, case-control studies are still typically used as initial validation methods for deep learning algorithms, as they are more convenient to perform and allow for establishment of a reference standard [23, 24].

**Figure 4:**
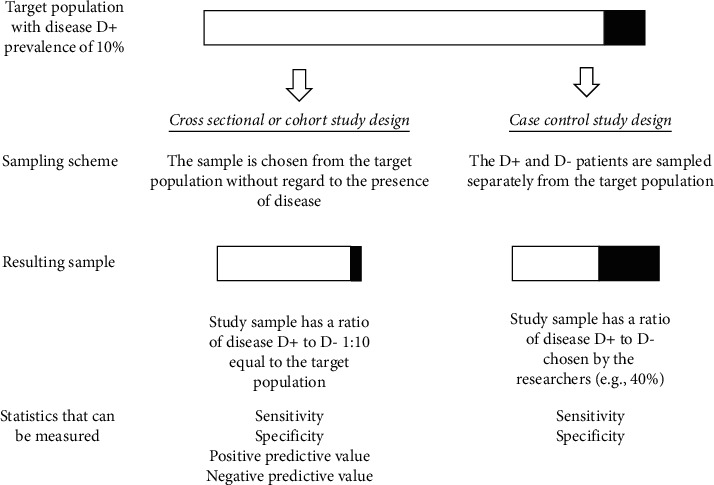
Cross-sectional and cohort designs allow for the calculation of a negative predictive value (NPV) and a positive predictive value (PPV), as they incorporate meaningful prevalence data. D refers to disease status with D+ meaning disease is present and D-patients meaning disease is absent.

Another limitation of the case-control design is that the positive predictive value (PPV) (probability that subjects with a positive test truly have the disease) and negative predictive value (NPV) (probability that subjects with a negative test truly do not have the disease) cannot be directly measured, as the ratio of cases to control is set by the investigator and disease prevalence is not reflected in the data (see Figure 4) [22].

## 4. Domain 2: Index Test

### 4.1. Information Bias

An important source of bias when evaluating the index test is the lack of blinding of the investigators to the results of the reference standard for each subject. Knowledge of the reference standard results may influence the interpretation of the index test results. This is also known as information bias. This type of bias can lead to larger deviations when the index test is not an objective measurement and depends on a rater's subjective assessment [25].

Aside from blinding to avoid information bias, it is important for diagnostic accuracy studies to prespecify the threshold used for the index test interpretation. A posteriori determination of a threshold in a data-driven way can lead to overestimation of test performance, especially in studies with a small number of subjects. This is because an optimal cutoff may be chosen based on the available results to favor overly optimistic measures of diagnostic accuracy [26].

For example, Kivrak et al. performed a study comparing computed tomography (CT) virtual cystoscopy with conventional cystoscopy for the diagnosis of bladder tumors, which they designed in a rigorous way to avoid introducing information bias. The authors report that the two experienced radiologists, who independently interpreted the virtual cystoscopy (the index test), were blinded to the findings of conventional cystoscopy (the reference standard). Additionally, the virtual cystoscopy was performed and interpreted prior to the conventional cystoscopy, thereby ensuring that the investigators were blinded to the results of the reference test [27].

### 4.2. Indeterminate Index Test Results

Patients with indeterminate or ambiguous results should not be excluded from the study, as this could limit the results to an unrepresentative spectrum of extremes and potentially introduce spectrum bias. In this case, it is preferable to transform the 2 × 2 table to a 3 × 2 table and report positive, indeterminate, and negative results separately. To ensure that diagnostic accuracy estimates are not overestimated, a conservative “intention to diagnose” approach should be followed; indeterminate cases that test positive with the reference test are classified as false negative for the index. Indeterminate cases that test negative with the reference test are classified as false positive for the index (see Table 2). In the scenario when the reference test also yields indeterminate results, the table may be extended to a 3 × 3 table to ensure transparent reporting [28].

A meta-analysis by Schuetz et al. [28] pooled coronary CT angiography studies to compare how the handling of nonevaluable results affects diagnostic accuracy estimates. As CT angiography interpretation can involve nonevaluable test results especially in areas with vessel calcifications [29], the authors can consider nonevaluable vessel segments as positive or negative, exclude them from analysis, or even exclude patients with nonevaluable segments altogether. The authors in this study found that handling the test results with an “intention to diagnose” approach using a 3 × 2 table yielded lower diagnostic accuracy measures (Area Under Curve 0.93) compared to the other approaches (Area Under Curve 0.96–0.99) [28].

## 5. Domain 3: Reference Standard

The reference test represents the gold standard to which the index test is being compared. The assumption is that the reference standard is 100% accurate, so any disagreement with the results of the index test is attributed to the limited sensitivity or specificity of the latter. However, reference standards that perfectly differentiate between patients with and without the target condition are rare and, thus, some patients will inevitably be misclassified [30].

### 5.1. Misclassification Bias

Misclassification bias, which is also called imperfect gold standard bias, occurs due to errors in the reference test. The reference test may be susceptible to errors either due to its interpretation or due to technical limitations. For example, an imaging exam can give erroneous results because of inexperienced readers or due to limited resolution. If pathology is used as a reference standard, sampling error is an additional factor which could lead to false-negative results. The effect of this bias on the diagnostic accuracy estimates can vary depending on whether the reference and index tests tend to err in the same direction on the same patients or the reference and index test errors are independent of each other. As a result, sensitivity and specificity can be over- or underestimated by this type of bias [22].

An example of misclassification bias can be found in a study by Ai et al. which determined the diagnostic accuracy of chest CT for the diagnosis of Coronavirus Disease 2019 (COVID-19). The reference standard was a Reverse Transcription Polymerase Chain Reaction (RT-PCR) test, which can give false-negative results in the early stages of the disease. The authors calculated the sensitivity of chest CT for the diagnosis of COVID-19 to be 97% and the specificity 25% but acknowledged in the limitations section that, due to misclassification bias, the sensitivity may have been overestimated and the specificity may have been underestimated by solely relying on the results of a single RT-PCR test [31].

Various methods have been proposed to correct for misclassification bias. One suggestion is adjusting the accuracy estimates based on external evidence about the degree and direction of the reference standard misclassification. Other ways to minimize this bias are to combine multiple tests to a composite reference standard or validate the index test usefulness by correlating directly with future clinical events or other clinical characteristics [32].

### 5.2. Diagnostic Review Bias

Another important consideration when evaluating the reference standard is whether it is interpreted without the knowledge of the index test results. A positive index test may drive raters to search the reference study more carefully for evidence of disease. This is known as diagnostic review bias [25]. As pointed out by Ransohoff et al. [20], an example of this bias can be found in a study by Meadway et al. [33] which evaluated the diagnostic performance of Doppler ultrasound compared to venography. No indication was provided that the venograms were examined independently of the Doppler studies and thus it is possible that knowledge of the Doppler results affected the venogram diagnoses.

### 5.3. Incorporation Bias

On some occasions, the index test may be part of the reference standard. The resulting bias is called incorporation bias and leads to overestimation of the sensitivity and specificity. Incorporation bias often occurs when the reference standard relies on clinical judgment as the clinician often uses the index test to arrive at a diagnosis. This bias will result in an overestimation of diagnostic accuracy [34]. An example of this bias in the radiology literature can be found in a study by Mater et al. which evaluated the diagnostic accuracy of shunt series radiographs and CT to assess for cerebrospinal fluid shunt malfunction. The clinical decision to proceed to shunt revision, which was used as the reference standard, was made by the neurosurgeons after reviewing the radiograph and CT imaging. Despite the introduction of incorporation bias, this decision was reasonable in this study due to the lack of an independent gold standard. The authors also acknowledged this concern in the limitations section by stating possible overestimation of the sensitivity [35].

## 6. Domain 4: Patient Flow and Timing

Diagnostic accuracy studies should be designed taking into account time-dependent changes of the disease on the studied population and follow—as much as possible—a homogeneous approach for all subjects. Intervals between the index and reference test and disturbances in the flow of the study, such as changes in the reference test or withdrawals, are important sources of bias [7].

### 6.1. Timing of the Index and Reference Test

The time interval between the conduction of the index and the reference tests should ideally be as short as possible. A long period between the two could lead to misclassification bias, as the disease might improve or deteriorate during the interval time. An interval of a few days could be reasonable for chronic diseases but would be problematic for acute diseases. For reference tests that require follow-up to determine whether the disease is present, an appropriate minimum follow-up time should be set for all patients [6]. For example, a systematic review investigated the diagnostic accuracy of MRI in the diagnosis of early multiple sclerosis using clinical follow-up as reference standard. The average follow-up period in the included studies ranged from 7 months to 14 years and the authors found that studies with shorter follow-up tended to overestimate the sensitivity and underestimate specificity [36].

### 6.2. Verification Bias

Verification bias is a form of bias introduced when not all patients receive the gold standard (partial) or when some patients receive a different reference test than the rest (differential) [3]. In partial verification bias, if the decision is made to perform the gold standard only for positive index test cases, the sensitivity will be overestimated (fewer false negatives) and specificity will be underestimated (more false positives) [37]. The effect of differential verification depends on the quality of the different reference tests that are being used. Using a superior reference test for the positive test results and a different reference test for the negative results will overestimate both sensitivity and specificity [3]. Notably, using the same gold standard for all patients may not be clinically or ethically appropriate. If verification bias cannot be eliminated by choosing a proper study design, it should be at least acknowledged or statistically corrected by the authors [38].

An example can be found in the Prospective Investigation of Pulmonary Embolism Diagnosis (PIOPED) study, which evaluated the diagnostic accuracy of Ventilation-Perfusion (V-Q) scan using conventional angiography as a reference standard. From the 131 patients with near normal/normal results on the V-Q scan, only 57 received angiography (gold standard). For the remaining 74, an alternative reference standard was used: no evidence of pulmonary embolism during one-year follow-up. The authors calculated that if those 74 patients were included in the analysis, the NPV for near normal/normal scan would have been 96% and if not, the NPV would have been 91%. So, they concluded that the true NPV value is somewhere between those two numbers but possibly closer to the first [39].

Another area in radiology where partial verification bias has been described is Single Photon Emission Computed Tomography (SPECT) for the diagnosis of coronary artery disease [40–43]. The decision to perform coronary angiography, which is the gold standard for the diagnosis of coronary artery disease, may be affected by the result of a preceding SPECT which introduces verification bias (also called posttest referral bias). Authors have utilized mathematical formulas (e.g., Begg and Greenes [44] and Diamond [45]) to adjust for this bias leading to significant changes in calculated diagnostic accuracy parameters. Miller et al. [42] reported an unadjusted sensitivity of 98% and specificity of 13% for SPECT in coronary artery disease. After correction with the Begg and Greenes formula, the sensitivity dropped to 65% and the specificity increased to 67% which indicates that verification bias can have an effect on accuracy estimation.

### 6.3. Attrition Bias

An important consideration is whether all patients were included in the analysis. Withdrawals lead to over- or underestimation of accuracy estimates (attrition bias) if the patients lost to follow-up differ in some way from those who remain. It is important for studies to report withdrawals and evaluate their effect on accuracy estimates [46]. An example of the effect of withdrawals on diagnostic accuracy estimates can be found in a study by Kearl et al. which investigated the accuracy of MRI and ultrasound in the diagnosis of appendicitis. Of the 589 patients included, the reference standards, which were pathology reports, surgical diagnosis, or clinical decision for medical treatment for appendicitis, were not available for 63 patients (10.7%) due to loss to follow-up. The authors acknowledged this limitation and analyzed the effect on diagnostic accuracy. A sensitivity analysis was performed, and the diagnostic accuracy was calculated separately as if all withdrawals were positive for appendicitis as well as if all withdrawals were negative for appendicitis with the reference standard [47].

## 7. Direction of Accuracy Measures due to Bias

Knowing the direction of diagnostic accuracy measures is a first step in countering the effect of bias in our interpretation of study results. The general direction towards which the diagnostic accuracy estimates may deviate can be predicted and depends on the specific type of bias. Rutjes et al. [16] and Lijmer et al. [3] quantified the effect of several study design biases on diagnostic accuracy measures (see Table 3). They used the Relative Diagnostic Odds Ratio (RDOR) as a parameter to compare studies with a specific methodological shortcoming to those without. An RDOR greater than one indicates that diagnostic accuracy parameters are overestimated in the study, while an RDOR less than one indicates that diagnostic accuracy parameters are underestimated in the study. The limitation with using RDOR is that important biases that have opposing effects on sensitivity and specificity may not cause significant directional changes in RDOR, which will remain close to one. This may be the explanation why both of these studies failed to detect statistically significant changes in the RDOR for some forms of bias [3] (see Table 3).

## 8. Conclusion

Diagnostic accuracy studies can suffer from many forms of bias. QUADAS-2 provides a useful framework for thinking about study design biases. Patient selection, index test, reference test, and patient flow/timing are the four main domains to be evaluated in each study, as they cover the primary sources of systematic error in diagnostic accuracy studies. Potential sources of bias should be acknowledged by the authors and their effect on test performance should be estimated and reported. We are encouraged to become familiar with the biases that can be found in diagnostic accuracy studies and critically assess the studies before applying the conclusions to their own clinical practice.

## Figures and Tables

**Table 1:** Types of bias in diagnostic accuracy studies and how to address them.

Bias type	How to address
Spectrum bias	Perform random or consecutive sampling; avoid excluding subjects with ambiguous results
Information bias	Implement blinding of the researchers to the results of the reference test when interpreting the index test; predetermine the thresholds when designing a study
Misclassification bias	Predict the direction and degree of deviation for the diagnostic accuracy in sensitivity analysis and adjust accordingly; create a composite reference standard
Diagnostic review bias	Implement blinding of the researchers to the results of the index test when interpreting the reference test
Incorporation bias	Address in the limitations section the possibility of overestimation of accuracy estimates and if possible, adjust accordingly
Verification bias	Use the same reference standard for all subjects and if not possible, acknowledge and measure the potential accuracy estimate error
Attrition bias	Study the characteristics of subjects lost and how they differ from those that remain; perform sensitivity analysis to calculate the range of diagnostic accuracy estimates as if all withdrawals tested positive or negative

**Table 2:** Various approaches for indeterminate index test results and their effect on sensitivity and specificity.

Indeterminate results	Sensitivity	Specificity
Excluded from analysis	Increased	Increased
Indeterminate results considered positive	Increased	Decreased
Indeterminate results considered negative	Decreased	Increased
“Intention to diagnose” approach	Decreased	Decreased

**Table 3:** Direction of diagnostic accuracy estimates by type of bias.

Type of bias	Sensitivity [3, 16, 22, 26]	Specificity [3, 16, 22, 26]	RDOR from Rutjes et al. [16]	RDOR from Lijmer et al. [3]
Sampling bias (consecutive over nonconsecutive sampling)	Increases if complex cases are excluded	Increases if complex cases are excluded	1.5, 95% CI (1.0–2.1)	0.9, 95% CI (0.7–1.1)
	Decreases if clear-cut cases are excluded	Decreases if clear-cut cases are excluded		
Spectrum bias	Increases when severe cases are overrepresented in the patient sample (“the sickest of the sick”)	Increases when healthy controls are overrepresented in the patient sample (“the healthiest of the healthy”)	4.9, 95% CI (0.6–37.3)	3.0, 95% CI (2.0–4.5)
Information bias: lack of blinding	Variable	Variable	1.1, 95% CI (0.8–1.6)	1.3, 95% CI (1.0–1.9)
Information bias: post hoc definition of cutoff	Increases	Increases	1.3 95% CI (0.8–1.9)	Not studied
Misclassification bias (imperfect gold standard)	Increases if errors in index and reference test are correlated	Increases if errors in index and reference test are correlated	Not studied	Not studied
	Decreases if errors in index and reference test are independent	Decreases if errors in index and reference test are independent		
Incorporation bias	Increases	Increases	1.4, 95% CI (0.7–2.8)	Not studied
Verification bias: differential (i.e., different reference standards)	Increases if the gold standard is used for positive index results and a different reference test (e.g., noninvasive and less expensive) is used for negative index results	Increases if the gold standard is used for positive index results and a different reference test (e.g., noninvasive and less expensive) is used for negative index results	1.6, 95% CI (0.9–2.9)	2.2, 95% CI (1.5–3.3)
Verification bias: partial	Increases	Decreases	1.1, 95% CI (0.7–1.7)	1.0, 95% CI (0.8–1.3)

RDOR: Relative Diagnostic Odds Ratio. CI: confidence interval.
